# Intensity- and Duration-Based Options to Regulate Endurance Training

**DOI:** 10.3389/fphys.2017.00337

**Published:** 2017-05-24

**Authors:** Peter Hofmann, Gerhard Tschakert

**Affiliations:** Exercise Physiology, Training and Training Therapy Research Group, Institute of Sports Science, University of GrazGraz, Austria

**Keywords:** exercise prescription, intensity, duration, endurance exercise, athletes

## Abstract

The regulation of endurance training is usually based on the prescription of exercise intensity. Exercise duration, another important variable of training load, is rarely prescribed by individual measures and mostly set from experience. As the specific exercise duration for any intensity plays a substantial role regarding the different kind of cellular stressors, degree, and kind of fatigue as well as training effects, concepts integrating the prescription of both intensity and duration within one model are needed. An according recent approach was the critical power concept which seems to have a physiological basis; however, the mathematical approach of this concept does not allow applying the three zones/two threshold model of metabolism and its different physiological consequences. Here we show the combination of exercise intensity and duration prescription on an individual basis applying the power/speed to distance/time relationship. The concept is based on both the differentiation of intensities by two lactate or gas exchange variables derived turn points, and on the relationship between power (or velocity) and duration (or distance). The turn points define three zones of intensities with distinct acute metabolic, hormonal, and cardio-respiratory responses for endurance exercise. A maximal duration exists for any single power or velocity such as described in the power-duration relationship. Using percentages of the maximal duration allows regulating fatigue, recovery time, and adaptation for any single endurance training session. Four domains of duration with respect to induced fatigue can be derived from maximal duration obtained by the power-duration curve. For any micro-cycle, target intensities and durations may be chosen on an individual basis. The model described here is the first conceptual framework of integrating physiologically defined intensities and fatigue related durations to optimize high-performance exercise training.

## Introduction

The regulation of endurance training is usually based on the prescription of individual exercise intensity zones/domains, (Meyer et al., 2005; Pescatello, 2014, p. 168) whereas, in contrast, exercise duration is rarely prescribed by individual measures and mostly set from personal experience or “usual” settings. Tremblay et al. (2005) critically mentioned that little research tempting to isolate the effect of exercise duration has been done but they suggested a duration threshold for hormonal responses especially for low intensity exercise. This is in line with earlier results by Viru et al. (1996) proposing that in exercise performed below a certain threshold intensity, hormonal responses will only occur after a certain long duration. As the hormonal changes trigger acute and chronic adaptation it is suggested that not only intensity but also the duration for any specific intensity is crucial to induce training effects or to avoid overload (Viru, 1995, p. 1–20). Recently, it was shown by Skovgaard et al. (2016) that muscle PGC-1α mRNA, identified as a key regulator of mitochondrial biogenesis and oxidative genes, did not change significantly after 60 min of endurance exercise in their study. This short duration and a high fitness level did not allow to sufficiently challenge muscle PGC-1α mRNA for the relative low exercise intensity (60% of VO2_max_) applied. However, including high intensity speed endurance exercise provided a stimulus for muscle mitochondrial biogenesis, substrate regulation, and angiogenesis.

Consequently, concerning training effects (Platonov, 1999; Noakes, 2000; Abbiss and Laursen, 2005), concepts integrating the prescription of both intensity and duration within one model are needed with respect to the main aims in endurance training which are to increase maximal oxygen uptake, the maximal sustainable speed, or power (threshold speed), and to increase economy and time to exhaustion (Lundby and Robach, 2015).

Several authors prescribed the distribution of various intensity domains for endurance training (Esteve-Lanao et al., 2005; Seiler and Kjerland, 2006; Seiler, 2010; Stöggl and Sperlich, 2015), but explicit prescriptions of an optimal duration for each individual intensity domain are still missing. Pettitt (2016) recently combined exercise intensity and duration by introducing a critical velocity similar to the critical power concept (Vanhatalo et al., 2011; Poole et al., 2016). The CP model itself is not based on physiological measures, although it seems to have a physiological basis which was shown to be related to the maximal lactate steady state intensity (Jones et al., 2008, 2010). This concept however, does not include a differentiation of all intensity domains (Meyer et al., 2005; Hofmann and Tschakert, 2010) which are known to trigger specific acute physiological responses, which are suggested to be crucial for a successful training adaptation (Hoppeler, 2016). Dekerle et al. (2003) as well as Pringle and Jones (2002) showed that the critical power calculated from a given range of exhaustion time did not correspond to the maximal lactate steady state (mLaSS) similar to Brickley et al. (2002) indicating the need to combine both aspects into one model recently shown by Burnley and Jones (2016).

It is well-prescribed that competitive endurance athletes using the polarization model train up to 13 training sessions per week with an intensity distribution of about 80% of total training volume performed at low intensity and about 20% high-intensity work such as interval training (Esteve-Lanao et al., 2005; Seiler and Kjerland, 2006; Seiler, 2010; Stöggl and Sperlich, 2015). From this point of view, a focus on optimization of both the low intensity-high volume and the high intensity-low volume parts of the training as well as concepts and models to prescribe both intensity and duration including physiologically relevant zones are required. Aim of the paper is to give a theoretical framework prescribing both intensity and duration for endurance training.

## Prescription of intensity

**Figure 1:**
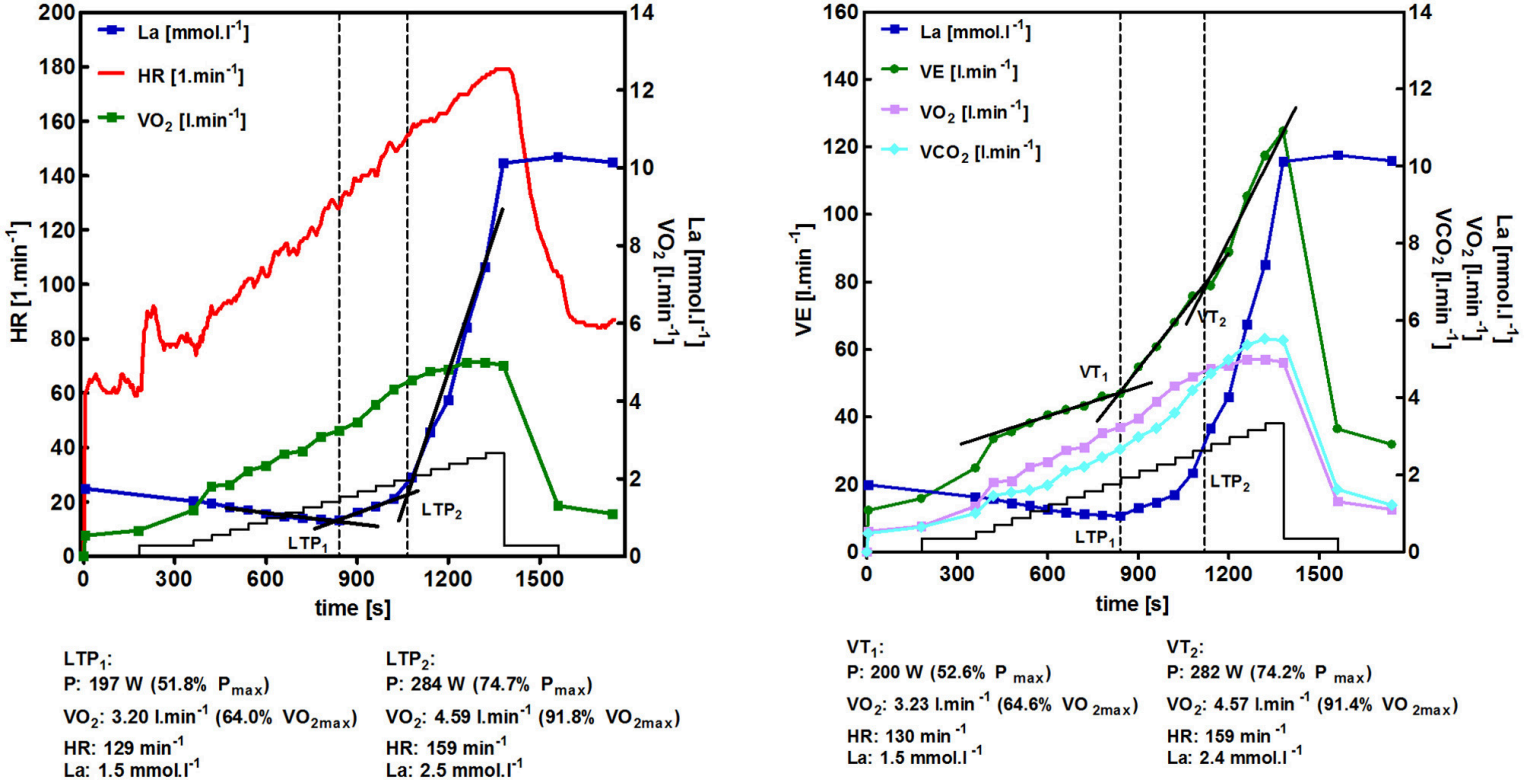
**Time course of heart rate (HR), oxygen uptake (VO_**2**_), and lactate (La) as well as the first (LTP_**1**_) and the second (LTP_**2**_) lactate turn point during an incremental cycle ergometer exercise test in a well-trained cyclist (A)**. Time course of ventilation (VE), oxygen uptake (VO_2_), carbon dioxide output (VCO_2_), and lactate (La) as well as the first (VT_1_) and the second (VT_2_) ventilatory turn point during incremental cycle ergometer exercise in a well-trained cyclist **(B)**.

The prescription of exercise intensity for endurance-type exercise is usually based on exercise markers from maximal and/or sub-maximal incremental exercise tests (Meyer et al., 2005). Guidelines recommend using percentages of maximal oxygen uptake (VO_2max_), maximal heart rate (HR_max_), or maximal power output (P_max_) for setting exercise intensity (Pescatello, 2014, p. 168). However, threshold or turn point concepts are suggested to be the gold standard for exercise intensity prescription in practice (Meyer et al., 2005) although still critically discussed (Mann et al., 2013). Actually, most authors agree to set training intensities by a three phase and two threshold model (Meyer et al., 2005; Hofmann and Tschakert, 2010) indicated by a first lactate (LT_1_/LTP_1_) or ventilatory (VT_1_) and a second lactate (LT_2_/LTP_2_) or ventilatory (VT_2_) threshold or turn point which has been successfully integrated into the practice (Seiler and Kjerland, 2006; Seiler, 2010; Algrøy et al., 2011; Muñoz et al., 2014a,b; Tønnessen et al., 2014, 2015). Figure 1 shows an example of the time course of selected variables and the according turn points LTP_1_/VT_1_ and LTP_2_/VT_2_ for a trained cyclist.

**Figure 2:**
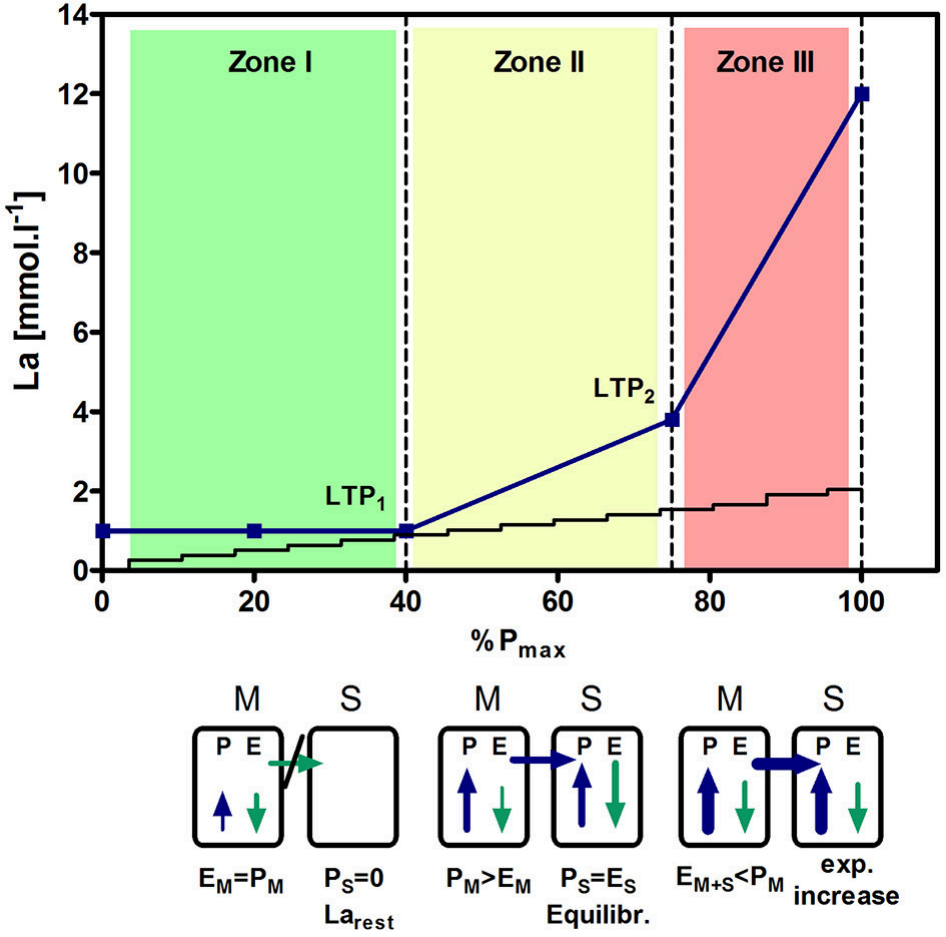
**Time course of blood lactate concentration (La) and first (LTP_**1**_) and second (LTP_**2**_) lactate turn points**. LTP_1_ is the first increase above baseline. Lactate production within the muscle (P_M_) is equal to the elimination rate within the working muscle (E_M_) and, consequently, no La has to be shuttled to the system (S). La within the system stays at resting level because the critical lactate clearance of the working muscle is not exceeded. This is the first zone which is metabolically balanced at muscle level (M) (E_M_ = P_M_). Above LTP_1_, blood La increases with increasing workloads. La production within the muscle (P_M_) exceeds the muscular rate of La elimination (E_M_) and La has to be shuttled to the system (S). Within the system, La from the muscle (P_S_) can be eliminated (by resting muscles, heart, brain,…) (E_S_ + E_M_ = P_M_) and La builds up an equilibrium (P_S_ = E_S_) at an elevated level, a so called lactate steady state on a systemic level. The critical La clearance rate of the system is not exceeded which gives a metabolically balanced situation (zone II). If the rate of La production within the muscle exceeds the maximal elimination rate of the system and the muscle (P_M_ > E_M_ + E_S_), La increases exponentially without a metabolically balanced situation (zone III). The transition from zone II to zone III is indicated by the LTP_2_.

Several variables enable to discern three distinct phases of metabolism and cardio-respiratory responses which allow setting defined intensities for continuous or interval-type exercise (Hofmann and Tschakert, 2010; Tschakert and Hofmann, 2013). According to the lactate shuttle theory (Brooks, 1986, 2009) the first lactate turn point (LTP_1_) is defined as the first increase in blood lactate concentration (La) accompanied by a first change of increase in ventilation (VT_1_) and distinct changes in other ventilatory variables. The second lactate turn point (LTP_2_) is defined as the second abrupt increase in La accompanied by a sharp increase in ventilation (VT_2_) and distinct changes in other ventilatory variables (Figures 1, 2). It has to be mentioned that the chosen incremental test protocol influences the accuracy of any threshold determination and the validity to prescribe constant load or intermittent-type exercise. A careful choice of the protocol is accordingly substantial. A detailed discussion of this problem, however, is not within the scope of this article but discussed elsewhere (McLellan, 1985; Amann et al., 2004).

### Continuous exercise

The first and the second turn points are sub-maximal markers from incremental exercise which can be used to prescribe defined exercise workloads with distinct and defined metabolic, cardio-respiratory, and hormonal responses as shown recently by our working group for constant load and matched intermittent-type exercise (Tschakert and Hofmann, 2013; Moser et al., 2015; Tschakert et al., 2015). During exercise below LTP_1_, no increase in La above baseline level was detected for constant load exercise, and it was shown recently that this intensity can be sustained for a very long duration of up to 24 h in trained ultra-distance athletes (Pokan et al., 2014). Increasing the workload above LTP_1_ leads to an increase in La above baseline, but after several minutes a La steady state is built up. The mLaSS is reached at LTP_2_ power output, but this intensity is clearly limited in time (Dittrich et al., 2014) but independent of exercise mode (Fontana et al., 2009), training status, and temperature (Périard et al., 2012). Although mLaSS intensity can be determined rather precisely, time to exhaustion at the mLaSS still can vary distinctively between athletes. Faude et al. (2017) showed a low reliability of time-to-exhaustion and blood lactate concentration at mLaSS indicating that a precise individual prescription of exercise still remains challenging especially with respect to duration.

**Figure 3:**
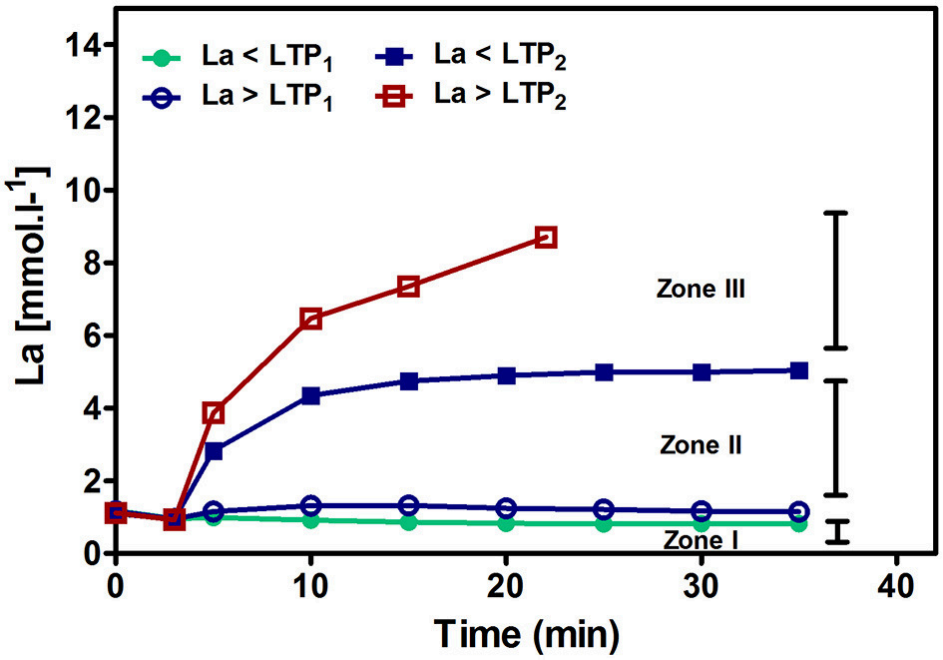
**Time course of blood lactate concentration (La) below and above the first (LTP_**1**_) and the second (LTP_**2**_) lactate turn points**. Intensities below LTP_1_ give a lactate steady state at resting level because the critical lactate clearance rate within the working muscle is not exceeded (zone I). For intensities above LTP_2_, some La produced within the muscle is shuttled to the system but La clearance is high enough to establish an equilibrium, the so called systemic La steady state (LaSS) (zone II). Just below LTP_2_ the maximal lactate clearance rate, the so-called maximal systemic LaSS is approached. Above LTP_2_, no equilibrium can be established and La increases with time with early termination of exercise due to non-sustainable acidosis (zone III).

Workloads above LTP_2_/VT_2_ lead to a constant increase in La until the individual level of tolerance has been reached. This is also reflected in the response of adrenaline and noradrenaline (Moser et al., 2015). Figure 3 shows schematically the time course of La for the three different exercise intensity zones. Percentages of maximal heart rate (%HR_max_), of maximal oxygen uptake (%VO_2max_), or % HR reserve (%HRR) as well as % oxygen uptake reserve (VO_2_R) are not able to discriminate these phases correctly on an individual basis (Hofmann et al., 2001; Meyer et al., 2005; Scharhag-Rosenberger et al., 2010).

Usually, the first and/or the second turn points (corresponding to the mLaSS) are applied to prescribe exercise intensity limits for prolonged endurance exercise training (Esteve-Lanao et al., 2005; Muñoz et al., 2014a; Tønnessen et al., 2014) whereas the first turn point is rarely investigated (Mann et al., 2013). It is obvious that LTP_2_/VT_2_ clearly discern between sustainable, metabolically balanced or non-sustainable, not metabolically balanced workloads, whereas during exercise near the first turn point a difference in acute responses can only be detected after a very long duration of exercise (Tremblay et al., 2005). Mostly, these small differences in intensity slightly below or above LTP_1_/VT_1_ are not detected and recognized by athletes although it might be important with respect to the particular maximal duration, grade of fatigue and the subsequent recovery time which has been described recently by Burnley and Jones (2016). These authors suggested distinct fatigue mechanisms for each intensity domain. A paucity of fatigue-related mechanistic studies was shown for the moderate and high-intensity domains but lee attention has focused on the low intensity part so far. As can be seen in Figure 3, an intensity slightly above LTP_1_ already increases La which indicates that the critical lactate clearance rate for the working muscle has been exceeded, and therefore, different hormonal and cardio-respiratory responses are suggested for this intensity level (Moser et al., 2015). In very prolonged exercise with blood lactate remaining at resting level throughout exercise it was prescribed that only after several hours fatigue occurs and increases with time until the limit of tolerance (Burnley and Jones, 2016). It has to be mentioned that energy stores play a substantial role regarding the maximal duration until the point of fatigue (Johnson et al., 2004).

### Interval exercise

The prescription of intermittent exercise is somehow more complicated compared to continuous exercise as the number of variables is higher. In addition to the workload intensity for the intervals (P_peak_), the total duration (t_total_) (number of intervals), the duration of the single workloads (t_peak_) as well as recovery intensity (P_rec_) and duration (t_rec_) and the corresponding mean load (P_mean_) have to be considered (Buchheit and Laursen, 2013a,b; Tschakert and Hofmann, 2013). Similar to constant load exercise (CLE), the mean intensity and total duration are main markers of the overall workload, but P_mean_ is influenced by the aforementioned variables with respect to the degree and the kind of fatigue and recovery (Burnley and Jones, 2016). Nonetheless, also for intermittent exercise, intensities (P_peak_, P_rec_, P_mean_) are suggested to be set in relation to sub-maximal (LTP_1_/VT_1_, LTP_2_/VT_2_) and maximal markers (P_max_) from an incremental exercise test: P_peak_ = P_max_, P_rec_ = %P_LTP1_, P_mean_ = %P_LTP2_ (Tschakert and Hofmann, 2013).

In addition, we could recently show that aerobic high-intensity interval exercise (HIIE) with short workload durations and P_mean_-matched constant load exercise produced similar acute metabolic, hormonal and cardio-respiratory responses (Moser et al., 2015; Tschakert et al., 2015). In contrast, HIIE with long workload durations but the same mean load yielded significantly higher acute physiological responses compared to short HIIE and CLE (Tschakert et al., 2015). This indicated that strictly planning interval-type exercise respecting all variables allows the regulation and the predictability of the acute physiological responses (Tschakert et al., 2015). In a recent paper we could show that even with a high-intensity running speed, short 10 s intervals, 20 s passive recovery but a very low mean load below LTP_1_, lactate levels were only slightly higher than resting level, and 30 min of exercise was clearly below the maximal duration which was, however, not obtained in this study (Wallner et al., 2014).

Similar to constant load exercise (Chidnok et al., 2012; Soares-Caldeira et al., 2012), the problem arises how to prescribe the optimal total duration (number of intervals) for intermittent exercise to identify optimal adaptation effects for any specific micro-cycle of a training period (Platonov, 1999; Lyakh et al., 2014). As long as there is a metabolically balanced situation (aerobic interval training), we may treat this problem similar to constant load exercise. In case of increasing La (anaerobic interval training), the optimal number of intervals may be set similar as it is performed in resistance-type exercise (Richens and Cleather, 2014). Again there is urgent need to identify any markers of optimal duration for both constant load and interval-type exercise. Burnley and Jones (2016) highlighted that the power-duration relationship exists not only for constant-power laboratory-based exercise, but also for variable-paced, self-paced, and intermittent or stochastic exercise, which more closely reflects the “real-world” athletic performance.

## Prescription of duration

**Figure 4:**
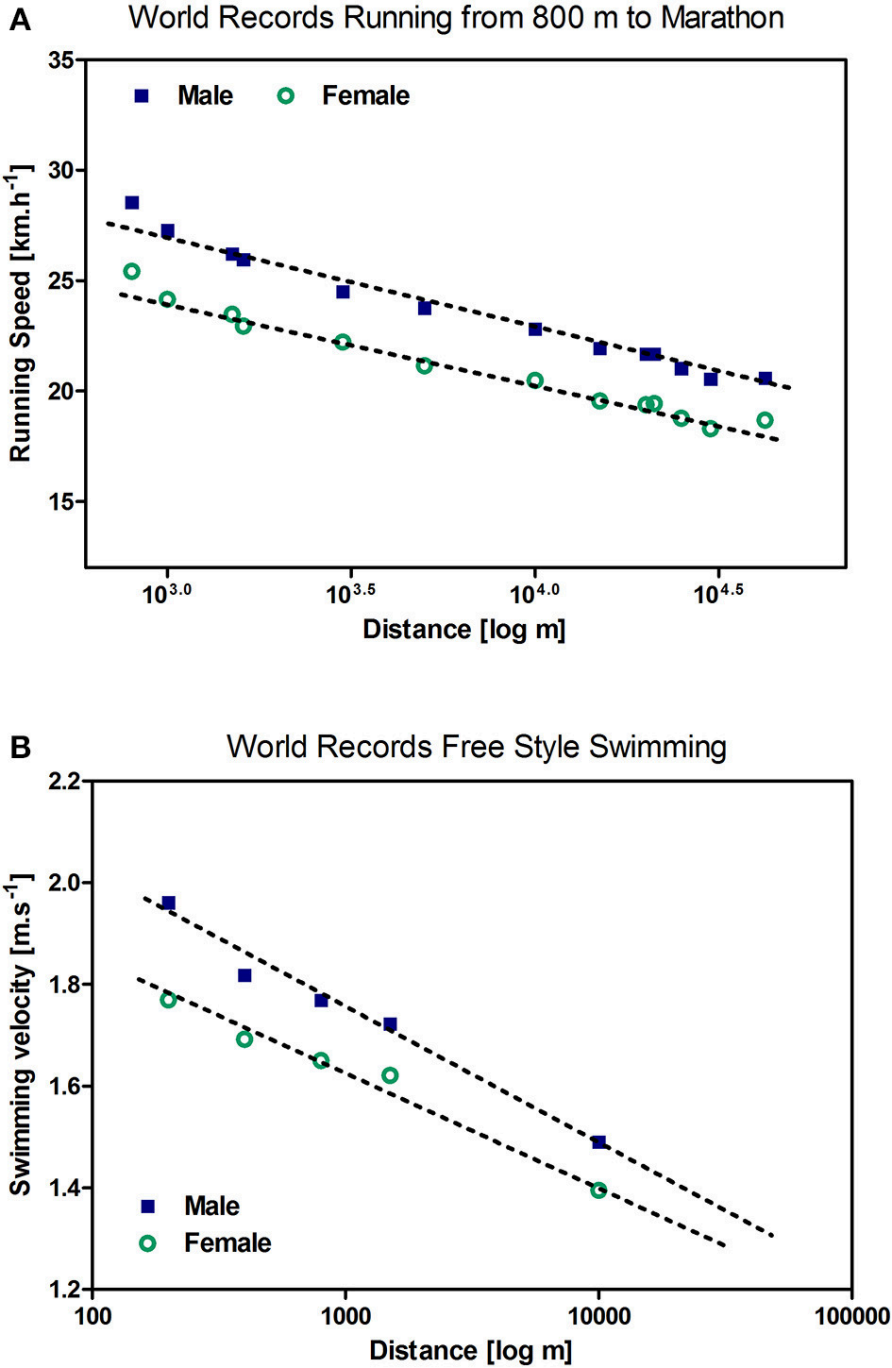
**Speed-duration relationship of endurance-type world records in running from 800 m to marathon race distance (A)** and swimming **(B)** was shown to be linear in a logarithmic scale for male and female athletes.

It is a fact that any certain intensity has its own critical time limit which is dependent on the type of exercise and the kind of athletes, but may be used as an individual diagnostic tool to prescribe exercise duration (Vanhatalo et al., 2011; Pettitt, 2016; Poole et al., 2016). Figure 4A shows the running speed for all endurance-type world records in continuous running from 800 m to marathon distance which has been described as a most perfect logarithm relationship for both men and women (Nikolaidis et al., 2017). A similar relationship can be shown for free style swimming (Figure 4B). The relationship between speed and distance is linear applying a logarithmic x-axis within this wide range of race distances. It is obvious that no speed-distance pairs above the linear line are possible (Burnley and Jones, 2016). This speed-distance or power-duration relationship can therefore be applied to detect the maximal speed or power output for any distance or duration but, no less importantly, to detect any maximal duration or distance for an arbitrarily chosen speed or power on an individual basis. Additionally, independent of the chosen intensity, this concept allows setting a targeted duration (% of maximal duration) for endurance exercise training with respect to improvement, maintenance or recovery purposes. To regulate these distances we apply the concept of Platonov (1999) who differentiated four domains of durations with selective adaptation (Viru, 1995, p. 251). This author suggested “very heavy maximal,” “heavy sub-maximal,” “moderate,” and “low” workloads with respect to duration, but independent of the chosen intensity (Table 1).

**Table 1:** **Definition of specific duration domains for endurance-type exercise (modified from Platonov, **1999**)**.

**Workload**	**Phase**	**Duration**	**Targets**
Low	1st phase of stable performance	15–20% of maximal duration until clear fatigue	Maintaining exercise performance and accelerated recovery
Moderate	2nd phase of stable performance	20–60% of maximal duration until clear fatigue	Maintaining exercise performance
Sub-maximal	Phase of compensated fatigue	60–75% of maximal duration until clear fatigue	Stabilization and moderate increases in performance
Maximal	Phase of clear fatigue	75–100% of maximal duration until clear fatigue	Distinct increases in performance

**Figure 5:**
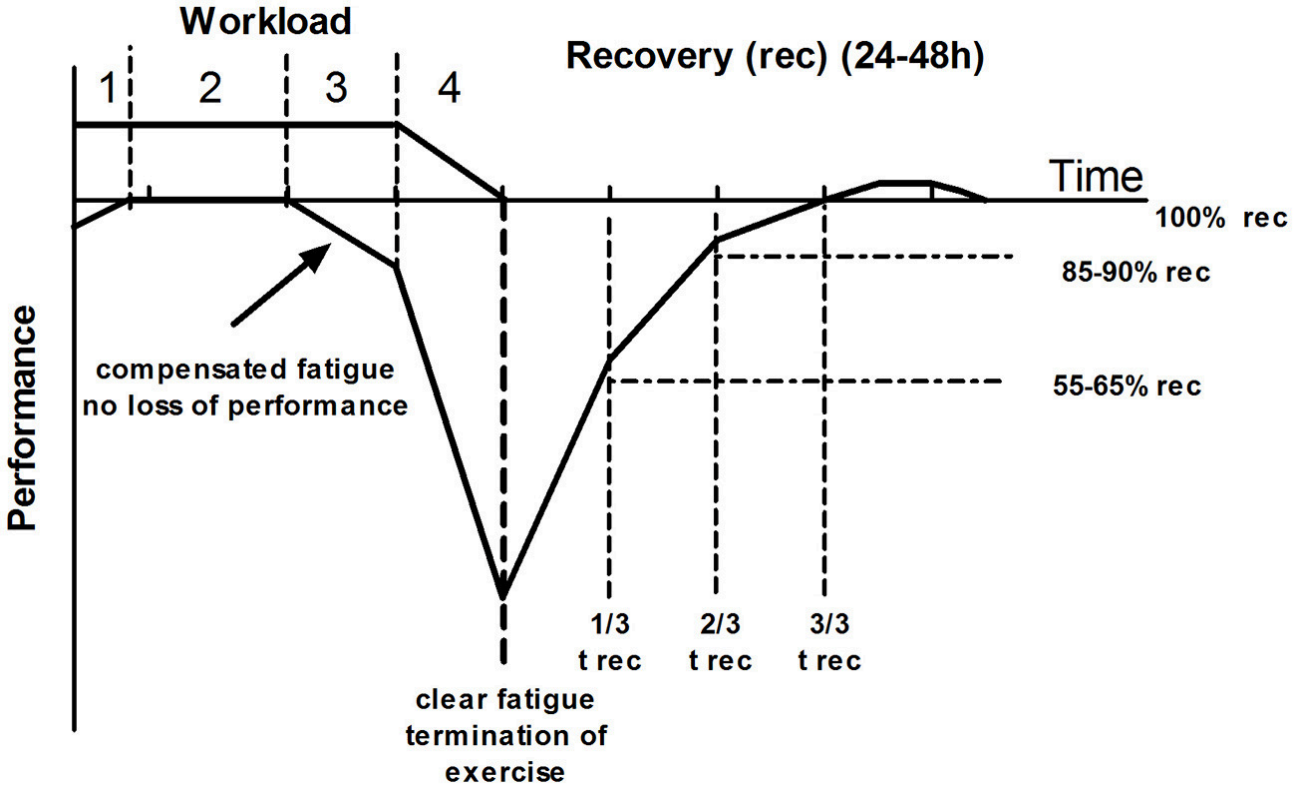
**Relationship between duration of exercise and fatigue (modified from Platonov, **1999**)**. There is a certain duration for each intensity leading to distinct grade of fatigue which terminates exercise (4), prolongs recovery times and increases “super-compensation” with increased performance. Reducing the duration to less than 75% of the maximal duration does not induce distinct but compensated fatigue without a loss in performance (3) and, consequently, a much shorter recovery time and less if any “super-compensation.” Reducing duration to less than 60% of the maximal duration (2) does not induce any fatigue and, therefore, does not increase performance, but rather stabilizes the given performance level. A duration less than 20% of the maximal duration is just a functional stimulation suggested adequate for regeneration.

**Figure 6:**
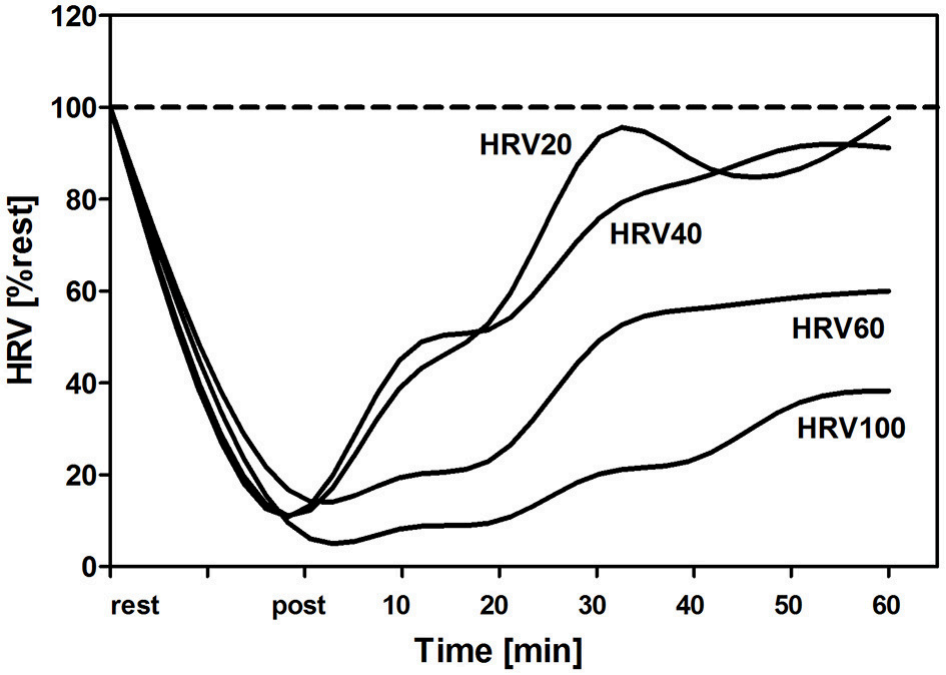
**Relative changes of heart rate variability markers (%HRV) during constant load exercise just below the second lactate turn point with different duration of the maximal sustainable distance (100%)**. It can be seen that recovery of the HRV is dependent on % maximal duration with early recovery at low to moderate distances (20, 40%) according to Platonov (1999).

Platonov (1999, p. 51) suggested that only “maximal exercise” (75–100% of the maximal duration until clear fatigue and loss of performance) induces distinct adaptation processes. This maximal exercise needs long recovery of about 48 h and longer but induces considerable performance increments (Kenttä and Hassmén, 1998; Issurin, 2009) (Figure 5) indicated by hormonal responses and signal-cascades yet not fully understood (Russell et al., 2013; Hoppeler, 2016; Kirby and McCarthy, 2016). Shortening the duration to 60–75% of the maximal duration until clear fatigue with the same intensity only leads to a compensated fatigue (some signs of fatigue which can be compensated without a loss in performance) which reduces recovery duration to half of the maximal exercise domain (about 24 h). The moderate workload is suggested between 20 and 60% of the maximal duration which does neither induce a compensated nor a clear fatigue and, therefore, does not increase performance but rather stabilizes it. Lastly, low workload defined as duration of less than 20% of maximal duration (again with the same intensity) induces regeneration and maintains exercise performance. Figure 6 shows an example of the recovery of heart rate variability (HRV) after maximal, sub-maximal, moderate, and low duration exercise with the same intensity applied (unpublished results). HRVwas shown to be sensitive for intensity and duration of exercise (Kaikkonen et al., 2010; Myllymäki et al., 2012).

**Figure 7:**
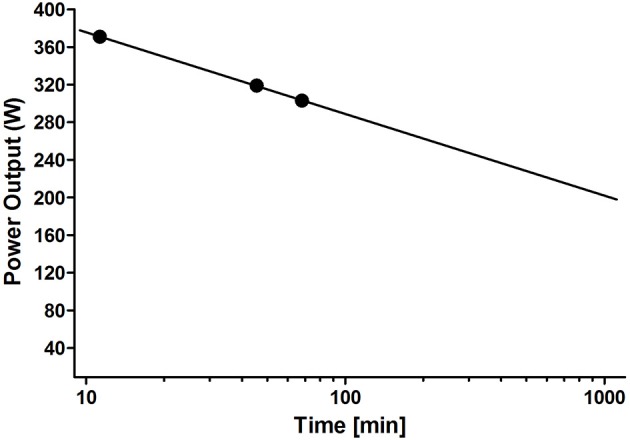
**Example of exercise intensity-duration relationship in a single well-trained cyclist**. As described in Figure 1, for any individual athlete, a plot of maximal speed and distance relationships can be drawn describing the maximal distance for any specific velocity or power output or the maximal velocity or power output for any chosen distance. According to the modified concept from Platonov (1999), these individual draws allow to prescribe the optimal distance (% of maximal duration) for selected intensities for various endurance-type training situations. To prescribe both intensity and duration on an individual basis, this concept needs to be combined to the usual sub-maximal performance diagnostic markers such as LTP_1_/ VT_1_ and LTP_2_/VT_2_.

From this concept (Platonov, 1999, p. 51; Viru, 1995, p. 251), we assume that for any single intensity within the three different intensity domains (Zone I: below LTP_1_/VT_1_; Zone II: between LTP_1_/VT_1_ and LTP_2_/VT_2_; Zone III: above LTP_2_/VT_2_), we may chose four different duration domains with clearly distinct adaptation effects on various physiological processes and exercise performance. To prescribe these duration domains one needs the maximal duration for at least 2–3 different intensities to draw the power-duration relationship as can be seen in Figure 7 which shows the same athlete as Figure 1.

### Model to combine the prescription of both intensity and duration

**Figure 8:**
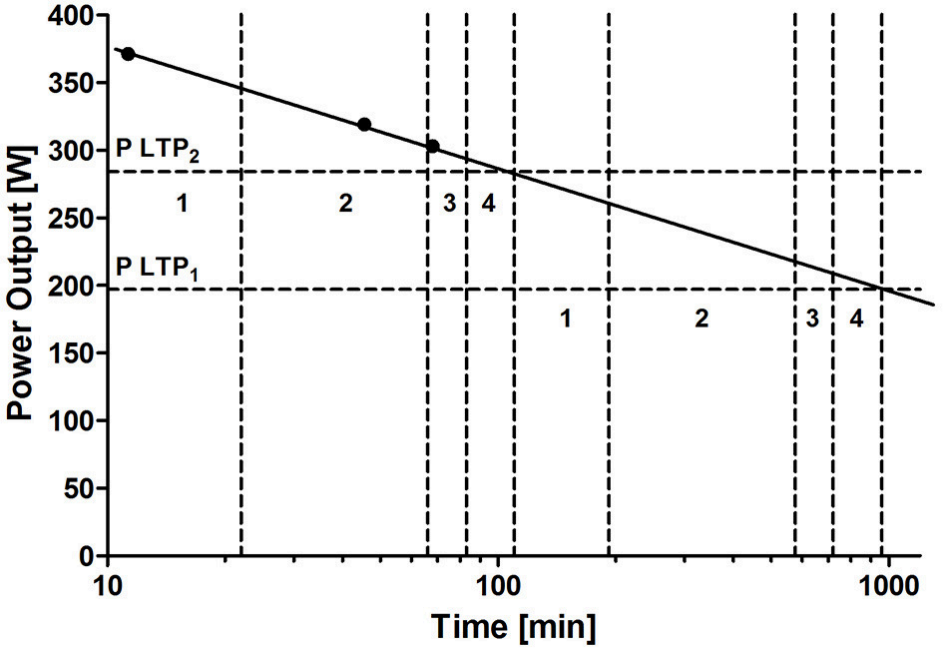
**Maximal and optimal (% of maximal) duration for exercise intensities at LTP_**1**_ and LTP_**2**_ in a well-trained cyclist applying the modified concept of Platonov (**1999**)**. For any specific metabolic, hormonal or cardio-respiratory target intensity (<LTP_1_/VT_1_; between LTP_1_/VT_1_ and LTP_2_/VT_2;_ > LTP_2_/VT_2_), the optimal duration with respect to the four workload domains “low” (1), “moderate” (2), “sub-maximal” (3), and “maximal” (4) may be derived from this graph of the maximal intensity-duration relationship.

Earlier approaches such as the model from Garcin and Billat (2001) applied a perceived exertion scale to attest both intensity and duration, but the optimal duration may not be obtained from this model. As can be seen in Figures 7, 8, the power output (or speed) to time (or distance) relationship allows discerning these specific durations for any intensity of interest by using four different duration domains according to Platonov (in (Viru, 1995), p. 251). As shown in Table 1, zone 1 is defined as low, zone 2 as moderate, zone 3 as sub-maximal, and zone 4 as maximal workload each producing different states of fatigue and, consequently, different effects of adaptation, which is in line with recent data from Tremblay et al. (2005). These authors showed a duration threshold for various hormone responses for a comparable low intensity of 50–55% VO_2max_ whereas a longer duration induced a favorable hormone profile which was suggested to support the mobilization of fuels for recovery and restoration of glycogen stores. In this combined exercise prescription model, the setting of work intensities should also be individualized and physiologically based by using turn point intensities as discerning markers for distinctly different metabolic, hormonal and cardio-respiratory responses (Tschakert and Hofmann, 2013; Moser et al., 2015).

This concept (Table 1 and Figure 8) enables athletes and coaches to fine-tune training volume and/or intensity to further optimize training processes which is of particular relevance when the limits of tolerance are reached. In addition, it allows a retrospective analysis of distances covered with given intensities in the past.

## Discussion and conclusions

The concept to combine turn point derived intensities and optimized durations may be specifically interesting with respect to the new polarized training concept (Seiler and Kjerland, 2006; Seiler, 2010; Muñoz et al., 2014a,b; Tønnessen et al., 2014) where 80–90% of training volume is set below LTP_1_/VT_1_ and up to 22% above LTP_2_/VT_2_ with very low volumes between both thresholds. It is, however, important to note that some authors also use fixed reference values for lactate such as 2 and 4 mmol.l^−1^ (Seiler and Kjerland, 2006; Guellich et al., 2009; Orie et al., 2014) which may overestimate the volume especially for the low intensity volumes. Our own results showed that La at LTP_1_ was found at 1.2–1.6 mmol.l^−1^ (Hofmann et al., 1997, 2001). An individual and accurate intensity prescription is crucial even at low power outputs near LTP_1_ since allowing intensities just 10% above LTP_1_ definitely shortens the time to clear fatigue by ~40% (Figure 8). As a consequence, high-volume training set above LTP_1_ may get too close to a fatigue state that avoids repeating high volumes on a regular daily basis. In addition, for low intensity exercise training, it is usually NOT intended to reach maximal duration (t_max_) but to apply a certain percentage of t_max_ (Table 1) in order to avoid fatigue and to guarantee the ability to repeat high volumes of training on a daily basis. However, we like to point out that dependent on the aim of a specific training period specific types of micro-cycles need to be structured combining exercise type, intensity, and duration.

Beside the attractiveness of the concept, several open questions and limits have to be addressed. Firstly, the chosen percentages of maximal duration are just marginally evidenced. To discern the low (regenerative) zone from a moderate zone without fatigue, a zone with compensated fatigue and finally zone 4 with clear fatigue needs to be taken with caution. Carefully conducted studies and retrospective analysis of distances covered at defined intensities are needed to identify the stability or variability of these percentages for athletes with different age, training status and sex, and individual physiological markers are needed to guide the training. As could be shown in a pilot test (Figure 6), heart rate variability might be a potential parameter to identify these cut-off points for duration as discussed recently (Kaikkonen et al., 2010; Saboul et al., 2016). Additionally, ratings of perceived exertion (RPE) scales may be helpful to identify these reference markers on a daily individual basis (Garcin and Billat, 2001; Seiler and Sjursen, 2004; Coquart et al., 2012).

A second important limit is the method to derive the power-duration or velocity—distance relationship. To obtain a valid regression line, data points must be obtained from highly motivated athletes from competitions. However, maximal performance changes during the training year due to periodization will make it a bit more difficult to obtain optimal distances throughout the year. Additionally, not all sports allow obtaining these markers under comparable and possibly standardized conditions such as in cycling or on-snow cross-country skiing. Semi-specific tests such as ergometer or ski-roller tests may help to overcome this problem.

A third limit may be the idea to maximize volumes by reducing intensity below LTP_1_/VT_1_. Although athletes may withstand such volumes from an acute metabolic state of view, some long-term problems such as orthopedic complaints (arthrosis, stress fractures) as well as disturbances in energy or fluid supply may arise from such as concept (Noakes, 2000; Cymet and Sinkov, 2006; Krampla et al., 2008; Weber, 2009; Warden et al., 2014).

Despite those limits, this concept gives a solid theoretical framework that allows optimizing both intensity and duration of the whole spectrum of endurance training load for the first time. It may help to improve exercise training for top level performance even though it is already close to the limits of tolerance for the human body.

## Ethics statement

This methodological consideration included a single pilot tests which were not part of a formal study but a proof of principle determination of markers from standard performance diagnostic tests which was performed in accordance with the recommendations of Declaration of Helsinki. The subject gave written informed consent in accordance with the Declaration of Helsinki.

## Author contributions

PH: Author wrote the manuscript, draw the figures, and graphs. GT: Author contributed equally in writing the manuscript.

### Conflict of interest statement

The authors declare that the research was conducted in the absence of any commercial or financial relationships that could be construed as a potential conflict of interest.
